# Comparison of mesenchymal stem cell sheets and chondrocyte sheets in a rabbit growth plate injury model

**DOI:** 10.3906/sag-1902-228

**Published:** 2020-06-23

**Authors:** Alper GÜLTEKİN, Yücel AĞIRDİL, Büşra Öncel DUMAN, Cansu Subaşı DEMİR, Erdal KARAÖZ

**Affiliations:** 1 Department of Orthopaedics and Traumatology, Derince Training and Research Hospital, Kocaeli Turkey; 2 Department of Orthopaedics and Traumatology, İzzet Baysal State Hospital, Bolu Turkey; 3 Stem Cell Department, Center for Stem Cell and Gene Therapies Research and Practice, Institute of Health Sciences, Kocaeli Turkey; 4 Department of Histology and Embryology, Faculty of Medicine, İstinye University, İstanbul Turkey; 5 Center for Stem Cell and Tissue Engineering Research and Practice, İstinye University, İstanbul Turkey; 6 Center for Regenerative Medicine and Stem Cell Research and Manufacturing,Liv Hospital, İstanbul Turkey

**Keywords:** Mesenchymal stem cell, chondrocytes, cell sheet, physeal arrest

## Abstract

**Background/aim:**

The treatment of posttraumatic deformities and differences in length between the extremities resulting from physeal injury remains controversial. The aims of this study were to compare the efficacy of tissue-engineered, monolayer, and allogeneic mesenchymal stem cell sheets and chondrocyte sheets for physeal arrest treatment and to investigate cell sheet technology as a novel method for cell transplantation in physeal cartilage repair.

**Materials and methods:**

A proximal tibial physeal injury was induced in New Zealand rabbits. Allogeneic mesenchymal stem cells (MSCs) and chondrocytes were cultured in temperature-responsive culture dishes and applied to the iatrogenic partial growth plate defects in single-sheet grafts (cell sheets). Treatment efficacy was determined using radiological measurements, as well as histological and immunohistochemical staining.

**Results:**

Treatment with MSCs and chondrocytes prevented endochondral ossification in the physeal plate, and bone growth resumed after treatment in both the MSC and chondrocyte cell groups. We found significant differences in radiological evaluations between pre- and posttreatment measurements in both MSC and chondrocyte groups. Transplanted cells were observed in the damaged area in both of the groups, which differentiated in the direction of growth plate cartilage.

**Conclusion:**

Our results support the hypothesis that MSC or chondrocyte transplantation using the cell-sheet technique described in the present study aids in the regeneration of cartilage tissue during physeal arrest after growth plate damage.

## 1. Introduction

Injury of the growth plate—the weakest region in the long bones of children—is a serious physical childhood trauma [1] that accounts for approximately 30% of childhood bone fractures [2]. Bony bridge formation in the growth plate and subsequent partial growth arrest may develop because of trauma-induced endochondral ossification and damage to cartilage formation. The treatment of secondary angular deformities and length differences between the extremities following an injury in growth plates remains controversial [3].

Recent studies have focused on cell-based treatments as the results of surgical methods (osteotomy, bone bridge excision, and subsequent placement of materials to inhibit bridge reformation) have been unsatisfactory [4–6]. While autologous chondrocyte transplantations have been attempted in animals, this technique was found to have resulted in major angular deformities and leg length discrepancies, as well as local immune-inflammatory reactions [7,8].

Other studies have concentrated on transplantation with mesenchymal stem cells (MSCs) owing to their multipotent properties. Allogeneic and autologous MSC transplantations have been compared in experimental models, where studies have investigated the effects of different types of scaffolds, as well as the ability of MSCs from different sources to migrate, differentiate, and proliferate [9–11]. However, there have been no reports that compare the clinical and histological results of transplanting MSCs derived from bone marrow versus chondrocytes in the treatment of physeal arrest. To the best of our knowledge, our study is the first to investigate and compare the superiority of the use of MSCs as opposed to chondrocytes. 

Recently, cell sheet engineering using temperature-responsive culture dishes has been developed as a novel alternative cell delivery method [12–15]. This technology involves stabilizing individually-dispersed cells until they grow into a thin, contiguous monosheet in which the cells communicate with each other and move together as a basic biological system that senses and responds to posttransplantation changes in physiological parameters. Thus, it helps to overcome common problems associated with current transplantation methods (e.g., scaffolds or single injection techniques), such as viability and problems with environmental adaptation.

Studies have been performed on the use of cells sheets in the treatment of both focal osteochondral defects and diffuse arthritis in joint cartilages [16–18]. However, for the first time, we attempted to develop a functional growth plate cartilage for the treatment of growth plate injuries using MSC and chondrocyte sheets that had been produced using temperature-responsive culture plates.

We hypothesized that the transplantation of MSCs or chondrocytes using cell sheet technology could enhance the regeneration of growth plate cartilage in proximal tibial physeal arrest in rabbits. The purpose of this study was to compare the ability of chondrocytes and bone marrow-derived MSCs to regenerate a functional growth plate in a rabbit tibia physeal injury model. We also aimed to investigate the efficacy of the cell sheet technique for MSCs and chondrocyte transplantation to treat physeal arrest in immature rabbits.

## 2. Materials and methods

### 2.1. Experimental design 

The laboratory animal protocol was approved by the Animal Ethics Committee of Kocaeli University. This study used 21 (10 males and 11 females) New Zealand white rabbits (≤6 week old, open growth plates, weighing between 550 and 700 g) obtained from the Experimental Animal Implementation and Research Centre of Uludağ University in Bursa, Turkey. Caregivers managed animal care and nutrition at the Experimental Animals Research and Application Unit of the university under the supervision of a veterinarian.

The rabbits were randomly divided into 3 groups with 6 rabbits each. Three other animals, which were not included in the experimental groups, were used as donors of both chondrocytes and MSCs. The medial part of the right proximal tibial physeal cartilage (5-mm diameter and 5-mm depth) was injured in all 18 animals, and the animals were subsequently observed for 4 weeks for bone bridge formation. Following bone bridge formation, the animals were subjected to a second surgical procedure to excise the bone bridge and were treated via the transplantation of cell sheets as follows: Group 1: transplantation with allogeneic chondrocyte cell sheets produced using temperature-responsive culture dishes; Group 2: transplantation with allogeneic MSC sheets produced using temperature-responsive culture dishes; and Group 3: no cell sheets transplanted after bone bridge excision.

Anesthesia was induced under a veterinarian’s supervision prior to all surgical procedures by applying intraperitoneal xylazine hydrochloride (5 mg/kg; Rompun 23.32 mg/mL; Bayer Türk Kimya Sanayi Ltd. Şti., İstanbul, Turkey) and ketamine hydrochloride (50 mg/kg; Ketalar 50 mg/mL; Pfizer, İstanbul, Turkey). The extent of anesthesia was determined by observing the palpebra, corneal reflex and tonus of the chin and skeletal muscles. Following the induction of anesthesia, the area of intervention was shaved and cleaned using povidone-iodine. Cefazolin (Zentiva Sağlık Ürünleri San. ve Tic. A.Ş., İstanbul, Turkey) was administered intraperitoneally at a concentration of 15 mg/kg to decrease the risk of infection. Normal physeal development was studied using the unoperated left tibias of each rabbit that had not undergone surgical procedures.

The rabbits were placed in cages in an environment maintained at 22 ± 2°C, on a 12-h light/dark cycle for 12 weeks to allow tolerable weight-bearing without any limitation of motion after the postsurgery recovery period. Following the operations, limping was observed for 7–10 days, after which the rabbits exhibited a hopping pattern for 2 weeks. After 3 weeks, all rabbits walked normally. No signs of infection were observed. The animals were euthanized 8 weeks after the second operation by administering an overdose of xylazine hydrochloride (Bayer Türk) and ketamine hydrochloride (Ketalar, 50 mg/mL, Pfizer).

Three rabbits that were not included in any of experimental groups were put under anesthesia for the isolation of MSCs and chondrocytes from the bone marrow that was aspirated from femoral condyle and iliac crest.

Isolation, culture, and characterization of rabbit bone marrow-derived MSCs (RbBM-MSCs) and chondrocytes were performed according to a previously published protocol, and MSCs were labeled using green fluorescent protein (GFP).

More information regarding these methods is provided in Karaoz et al. [19,20]. As chondrocytes are somatic cells with a relatively low replication rate and the expression of fluorescence could not be detected, GFP labeling was applied to only the MSCs in this study.

### 2.2. Culture of MSCs and chondrocyte sheets ontemperature-responsive culture dishes and collection as a uniformed plaque

**Figure 1:**
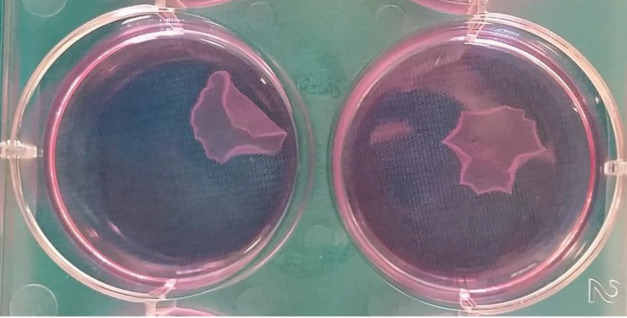
Chondrocyte and mesenchymal stem cells (MSC) sheets obtained in temperature responsive culture dishes. After proliferation for 1 day on temperature-responsive dishes, MSCs and chondrocytes were transferred to separate incubator set at 20 °C for 30 min to induce spontaneous detachment of the cell sheets.

Six-well (35-mm) Upcell (Upcell, CellSeed Inc., Tokyo, Japan) dishes—which are composed of a temperature-responsive polymer, poly (N-isopropylacrylamide)—were used to culture isolated cells. Mesenchymal stem cells and chondrocytes were seeded at a density of 4×106 cells/dish onto each 35-mm temperature-responsive culture dish. Overconfluent MSCs and chondrocytes on the temperature-responsive dishes were transferred to separate incubator (at 20 °C) for approximately 30 min, causing the MSC and chondrocyte sheets to spontaneously detach (Figure 1).

### 2.3. Preparation of transplantable cell sheets

All traces of culture medium were aspirated from temperature-responsive dishes, cells were rinsed twice with PBS, 500 µL of PBS was added into the dishes, and a polyvinylidene difluoride (PVDF) membrane (Thermo Scientific Nunc Upcell 35-mm dish, ref. no. 174914) was placed on the top of the cell sheets in the dishes to serve as a support membrane for transplantation into the damaged physeal area [21]. 

### 2.4. Induction of physeal injury

**Figure 2:**
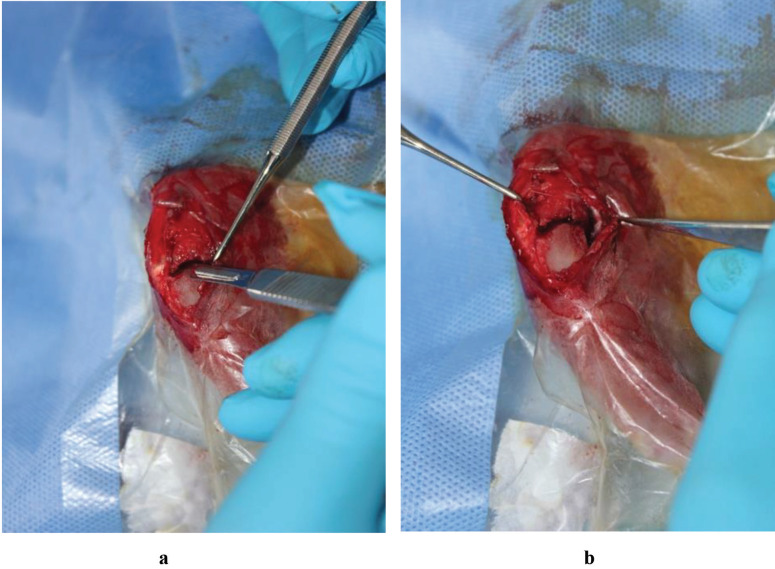
Induction of physeal injury to proximal medial physis. (a) Damage of proximal medial physis using a scalpel. (b) Proximal tibial medial half of physis after injury.

Under the appropriate surgical aseptic conditions and general anesthesia, the right knee of each rabbit was penetrated by making an anteromedial longitudinal incision. The joint capsule was opened, and the proximal tibial epiphysis was exposed. The medial part of the physeal cartilage (5-mm diameter and 5-mm depth) was injured using a curette and excised using a No. 11 scalpel (Figures 2a and 2b). Subsequently, the joint was irrigated with saline, the joint capsule was sutured using 4/0 polyglactin (Vicryl; Ethicon Inc., Johnson & Johnson Company, Somerville, NJ, USA), and subcutaneous tissue and skin were sutured using 4/0 polypropylene (Prolene; EthiconInc., Johnson & Johnson Company).

Postoperative radiological imaging was performed on day 1 and weeks 4 and 12 (i.e. the maximum duration of the study). After radiological observation of bone bridge formation at week 4, the animals were subjected to a second surgical procedure for bone bridge excision and the transplantation of MSC and chondrocyte cell sheets.

### 2.5. Bone bridge excision and transplantation of cell sheets

**Figure 3:**
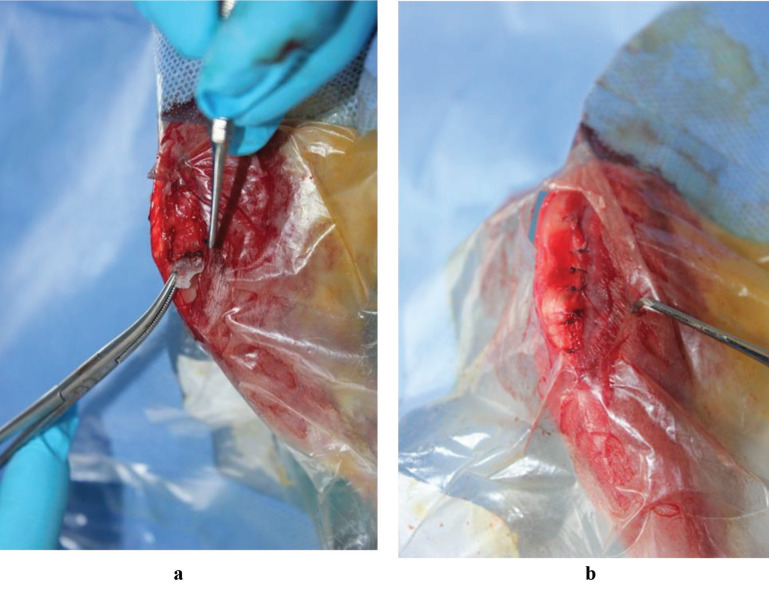
(a) Application of cell sheets after the excision of the bone bridge owing to physeal arrest. (b) Repair of skin following closure in anatomic sheets.

Under the same aseptic surgical conditions, the joint capsule was opened through the previous anteromedial incision, and the previously damaged area of the proximal tibial epiphysis was exposed. The bone bridges that had formed were excised using a No. 11 scalpel and a 1.1-mm drill (Colibri, Synthes, Oberdorf, Switzerland). Subsequently, a piece of PVDF sheet with an MSC or chondrocyte cell sheet was placed on the back of the injury site (Figure 3a). The PVDF support membrane was drawn back slowly with forceps. Lastly, the periosteum, subcutaneous tissue and skin were closed in anatomical layers (Figure 3b).

**Figure 4:**
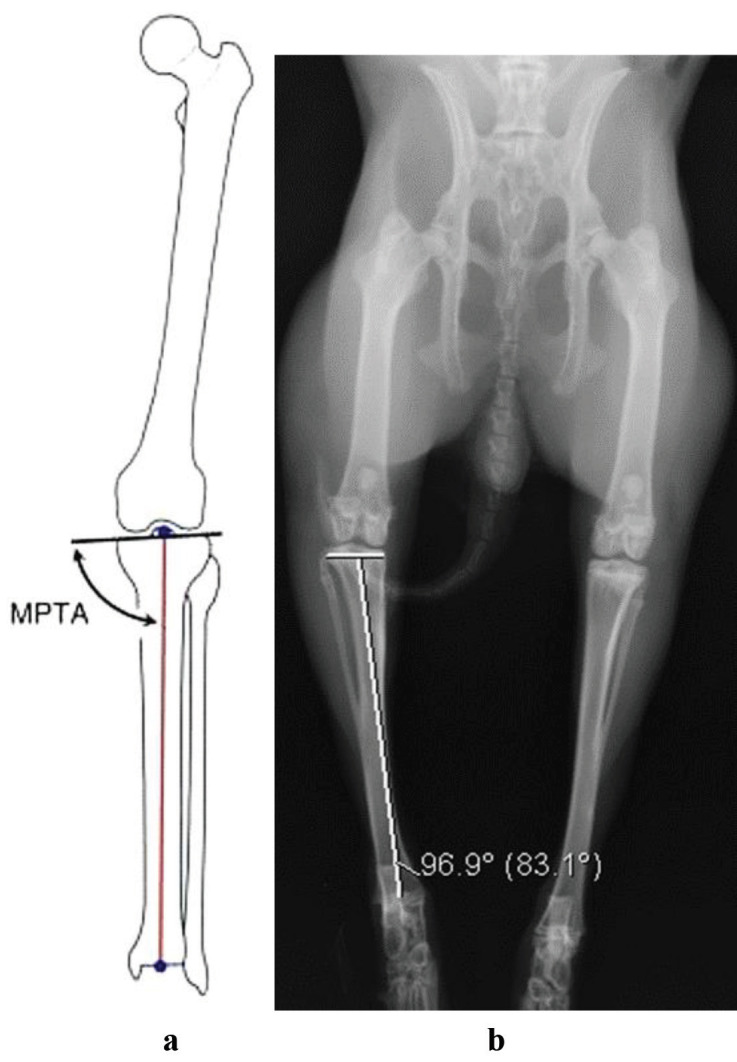
Measurement of the medial proximal tibial angle (MPTA). (a) Schematic diagram and (b) digital measurement on radiographs.

Plain radiographs were obtained using Digital Imaging and Communications in Medicine (DICOM) software to determine differences in length between operated and nonoperated tibias, tibiofemoral angles, and varus/valgus deformities. Tibial length was determined by measuring the distance between the highest point of the lateral plateau of the tibia and the middle point of the tibial plafond. The medial proximal tibial angle (MPTA) was defined as the angle between the tibial anatomical axis line and the tibia plateau line (Figures 4a and4b).

The tibias of all animals (n = 18) were evaluated in terms of the structural properties of physeal cartilage and for observations of possible transplant rejection (including lymphocyte infiltration and cartilage separation) using hematoxylin and eosin (H&E), immunohistochemical, and immunofluorescent staining.

The differentiation of transplanted MSCs was evaluated by a staining of the phosphorylated proteoglycans in the cartilage matrix with alcian blue (Atom Scientific, Manchester, UK) and safranin-O. Immunohistochemical and immunofluorescent staining was performed for the proteins aggrecan and Sox-9 (as described below), which are involved in mesenchymal differentiation into chondrocytes.

The ready-to-use Ultravision Detection System Large Volume Anti-Polyvalent HRP immunohistochemistry kit (Thermo Fisher Scientific, Cheshire, UK) was used according to the manufacturer’s protocols. Sections were stained to study antigen expression using monoclonal primary antibody for aggrecan (SC-33695) (Santa Cruz Biotechnology, Dallas, TX, USA) and polyclonal antibody Sox-9 (AB-5535) (Chemicon International Inc., Temecula, CA, USA). After staining, tissue sections were covered with immunohistomount sc-45086 (Santa Cruz Biotechnology) mounting medium and analyzed using a light microscope (Zeiss Z1 Axio Observer; Carl Zeiss Iberia, S.L., Spain). PBS was used as a negative control instead of primary antibodies using the aforementioned protocol. An immunohistochemical staining protocol was used to detect MSCs that were positive for GFP using an anti-GFP antibody (Sc-5385; Santa Cruz Biotechnology) as previously described [22]. The cells were subsequently examined using fluorescence microscopy (Zeiss Z1 Axio Observer). 

### 2.6. Statistical analysis

Statistical analyses were performed using SPSS Version 20.0 (IBM Corp., Armonk, NY, USA). Normally-distributed data were evaluated using the Kolmogorov–Smirnov test. Numerical variables were expressed as a mean ±standard deviation, median (25th–75th percentile), and frequency (%). Differences in numerical variables with normal distribution among the groups were analyzed using one-way analysis of variance and Tukey’s post hoc test, while differences in numerical variables with nonnormal distribution were analyzed using the nonparametric Kruskal–Wallis test and Tukey’s post hoc test. Numerical data with nonnormal distributions (leg length and deformity measurements) were evaluated using the Wilcoxon signed-rank test. Statistical significance was determined at P < 0.05. 

## 3. Results

### 3.1. Flow cytometry 

**Figure 5:**
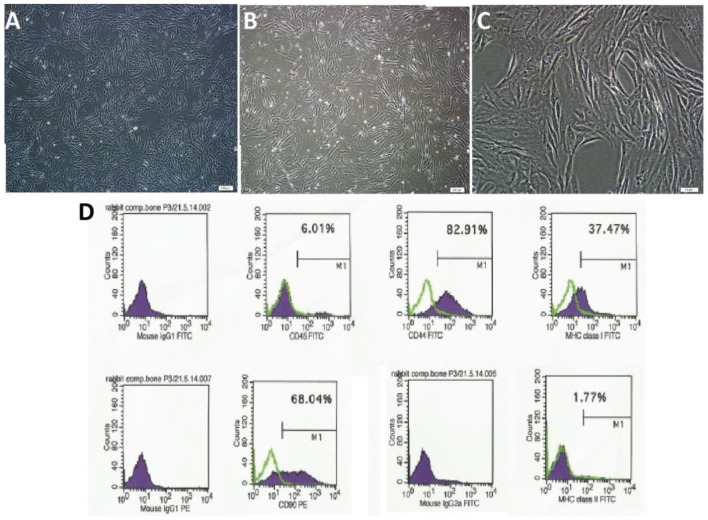
The cross-phase microscopic images of the rabbit bone marrow-derived cells (RbBM-MSCs) derived from the rabbit bone marrow and analyses of immunophenotypic characteristics of MSCs by flow cytometry. (a) Passage 1 (P1), day 4; (b)Passage 2 (P2), day 1; (c) Passage 3 (P3), day 3. Scale bars: a and b: 200 μm;c: 50 μm; (d) Flow cytometry data showing the immunophenotypic characteristics of MSCs isolated from rabbit bone marrow (P3) using CD45, CD90, and CD44 MHC class I and MHC class II markers are shown in (d).

Following 3–4 days of incubation, RbBM-MSCs proliferation started, and the cells gradually grew into small colonies (Figures 5a–5c). Flow cytometry analyses revealed that RbBM-MSCs were positive for CD90, CD44 MHC class I. Although a small fraction of positivity was observed for CD45 and MHC class II, 6.01 % and 1.77 %, respectively (Figure 5d), they were accepted as negative for CD45 and MHC class II.As explained and detailed further in the discussion section, this small fraction of heterogeneity was considered to have resulted due to changes in the phenotypic properties of MSCs originating from rabbit bone marrow during the culture process.

### 3.2. In vitro differentiation of RbBM-MSCs

**Figure 6:**
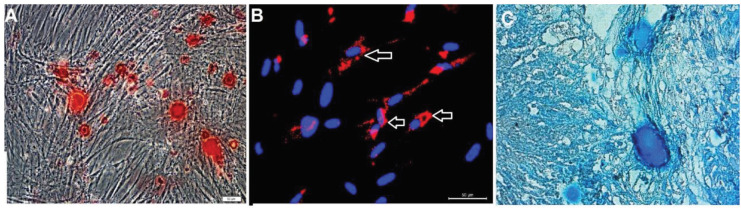
(a) Calcium deposition in the extracellular matrix stained with red after osteogenic differentiation (Alizarin Red-S) 29th day (scale bar: 200 μm);(b) Rabbit bone marrow-derived cells (RbBM-MSCs, passage 3) were fixed with oil red O after 18 days of culture. Differentiation in experimental rabbits is confirmed by the staining of oil droplets by oil red O and immunofluorescence staining. The arrows indicate the neutral lipid vacuoles stained with oil red O (scale bar: 50 μm); (c)This image shows chondrogenic differentiation via alcian blue staining after 30 days of culture (scale bar: 50 μm).

In the osteogenic differentiation, cells proliferated and reached almost complete confluency after 8–10 days of incubation. Later, the cellular aggregates were observed in differentiated cultures and gradually increased. The aggregates were characterized by the presence of amorphous material deposits. These nodular aggregates in osteogenic cultures were stained with Alizarin Red S after 29 days, demonstrating that the amorphous deposits were actually calcium deposits (Figure 6a).

When RbBM-MSCs were fixed with Oil Red O after 18 days of culture, the oil vesicles that had formed in the experimental group exhibited a red color, indicating differentiation (Figure 6b). In the cells, lipid droplets enlarged and invaded the entire cytoplasm.The lipid droplet formation was not observed in undifferentiated RbBM-MSCs. Chondrogenic differentiation was performed using a micropellet culture technique. Cell differentiation in the paraffin-embedded sections was determined histochemically by staining with alcian blue (Figure 6c).

### 3.3. Cell cultureand immunofluorescence stainingof chondrocytes

**Figure 7:**
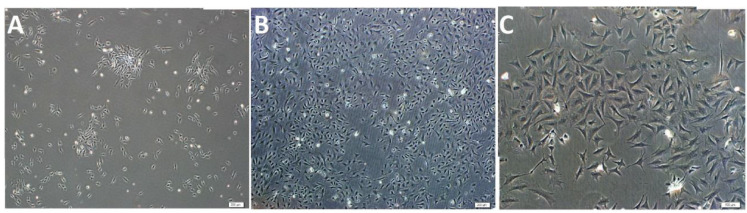
Contrast-phase microscopic images showingthe appearance of chondrocytes from rabbit cartilage tissue in culture. (a) Passage 1 (P1), 3 d; (b) P1, 7 d; (c) Passage 3 (P3), 4; (d) Chondrocyte colonies are observed in the first days of primary culture (day 3) in panel:a. Scale bars; a and b,200 μm; c: 50 μm.

**Figure 8:**
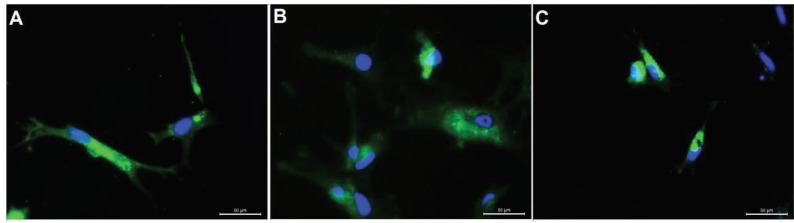
Images of immunofluorescence staining used in the characterization of chondrocytes from rabbit articular cartilage.For different areas on the same slide: a, b, and c: Collagen II (green), DAPI (blue). The nuclei were labeled with DAPI (blue) (scale bars: 50 μm).

We observed that chondrocytes exhibited a star-shaped morphology and were prone to proliferation by forming colonies in the first passages of culture. Following 3–4 days of incubation, we observed that the growth capacities of the cells increased (Figures 7a and 7b) and that the passage times were 4–5 days with a coating of 90% of the culture dish (Figure 7c). In immunofluorescence studies, expressions of chondrocytes cell markers were positive for collagen II (Figures 8a–8c).

### 3.4. Radiological observations

The lengths of the tibias were measured, and differences between the right (operated) and left (nonoperated) tibias were calculated, which yielded a leg length discrepancy. Although length differences existed between the operated and nonoperated tibias in all 3 groups at the end of the study, we observed a significant intergroup difference (P < 0.05; Table).

**Table:** Length and de

	Right tibia (mm)	Left tibia (mm)	MPTA right (degree)	MPTA left (degree)
Chond#1	105	105	75	87
Chond#2	101	103	93	89
Chond#3	97	101	67	87
Chond#4	102	104	76	86
Chond#5	101	104	85	86
Chond#6	101	106	83	88
MSC#1	105	111	82	83
MSC#2	110	110	79	81
MSC#3	90	109	58	85
MSC#4	97	107	63	85
MSC#5	104	111	67	86
MSC#6	91	108	60	83
Control#1	102	106	70	86
Control#2	88	105	45	82
Control#3	89	105	49	78
Control#4	94	105	49	76
Control#5	89	102	60	79
Control#6	89	92	52	94

Chond: Chondrocyte group; MSC: Mesenchymal stem cellgroup; MPTA: Medial proximaltibial angle.

The measurement of MPTA at week 12 in both knees of the rabbits was used to compare the normal growth of the contralateral tibia, and differences between both sides (right and left) were calculated. An increased difference in the angle indicated an increase in varus deformity, which is an indicator that treatment was not successful. The distribution of differences in MPTA angles measured at the proximal part of the right and left tibias was uneven between the groups (P < 0.05) after week 12. A significant difference was found between the chondrocyte and control groups (P = 0.006), while no significant difference was found between the MSC and control groups (P = 0.13). The difference in the chondrocyte group was smaller than that of the control group, revealing normal physeal development with minimal angular deformity (Table).

### 3.5. Histological and immunohistochemical assays

**Figure 9:**
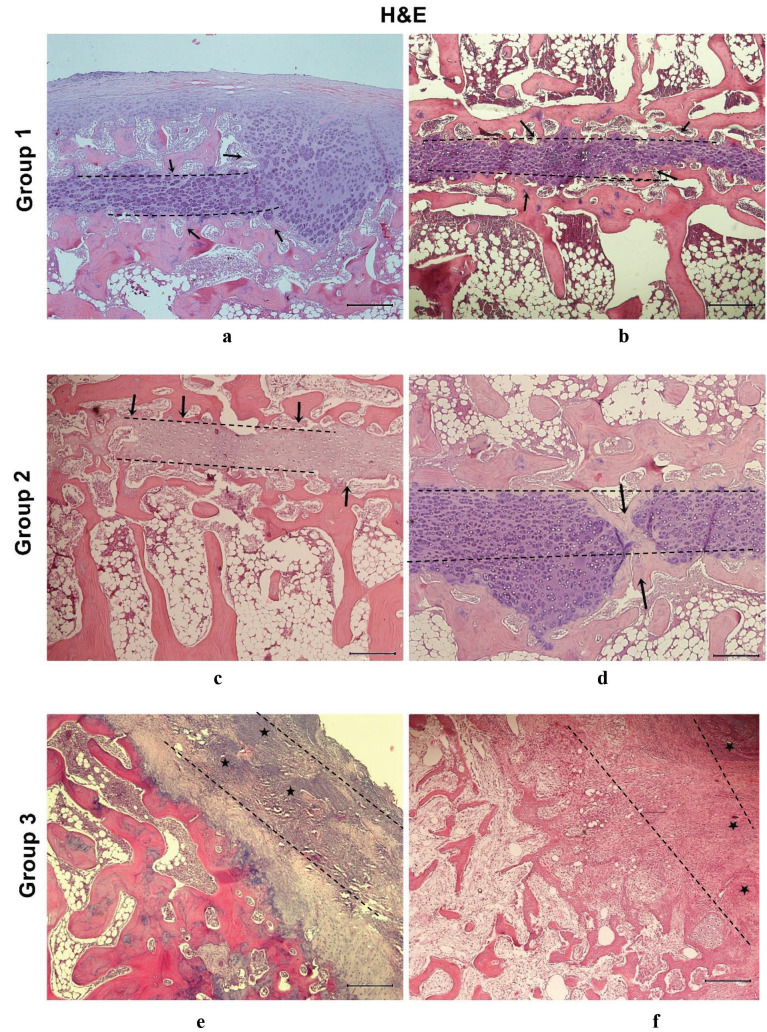
Hematoxylin and eosin (H&E) stained sections from all 3 experimental groups. (a) H&E staining of sections from Group 1, in which proximal tibial physeal injury was induced, and a chondrocyte cell sheet was applied, revealing that the transplanted chondrocyte cell sheet was located between the physis and articular cartilage; (b) Bone bridge formation (black arrow) was prevented in the injured area; (c) H&E staining of sections from Group 2 revealed that the transplanted mesenchymal stem cell (MSC) sheet was located in the damaged area and prevented the formation of a bone bridge (black arrows); (d) The stained sections of the same damaged area of another rabbit revealed that the MSC cell sheets prevented pathological processes, such as fibrous tissue formation from to damage of the physis; (e) H&E staining of the sections from control rabbits (Group 3: proximal tibial physeal injury was induced and no treatment was applied) revealed the formation of fibrous tissues (indicated by black stars); (f ) Pieces of cartilaginous tissue can also be seen in the damaged area (indicated by stars). The transverse physeal injury line is indicated with a dashed line in all images (scale bar: 500 μm)

**Figure 10:**
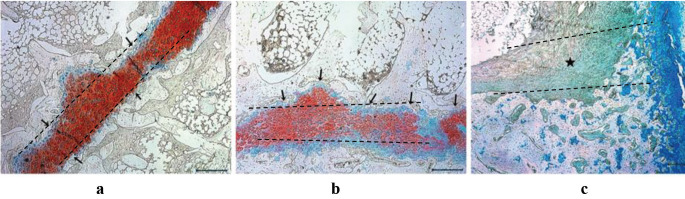
Alcian blue staining of a section from each experimental group. (a) Alcian blue staining of a section from Group 1, in which proximal tibial physeal injury was induced, and a chondrocyte cell sheet was applied. Staining revealed chondrocytes located on the growth plate (black arrows), which prevented bone bridge formation; (b) In Group 2, a transplanted mesenchymal stem cell (MSC) sheet (black arrows) prevented bone bridge formation and differentiated chondrogenically, facilitated by the microenvironment; (c) In Group 3, fibrous tissue formation (black star) was observed. Scale bars: 500 μm. The transverse physeal injury line is indicated by a dashed line in all images.

H&E staining of the paraffin sections obtained from Groups 1 and 2 animals showed that the transplanted cells were located between the epiphysis and joint cartilage, thereby preventing the development of bone bridge tissue (Figures 9a–9d). However, the evaluation of the corresponding sections in Group 3 revealed fibrous tissue and bone bridge formation, suggesting growth plate arrest (Figures 9e and 9f). Similarly, while epiphyseal cartilage was preserved in sections from Group 1 (Figure 10a), diffuse fibrous tissue and bone bridge formation were observed in tissue samples from Groups 2 and 3, following staining with alcian blue (Figures 10b and 10c).

**Figure 11:**
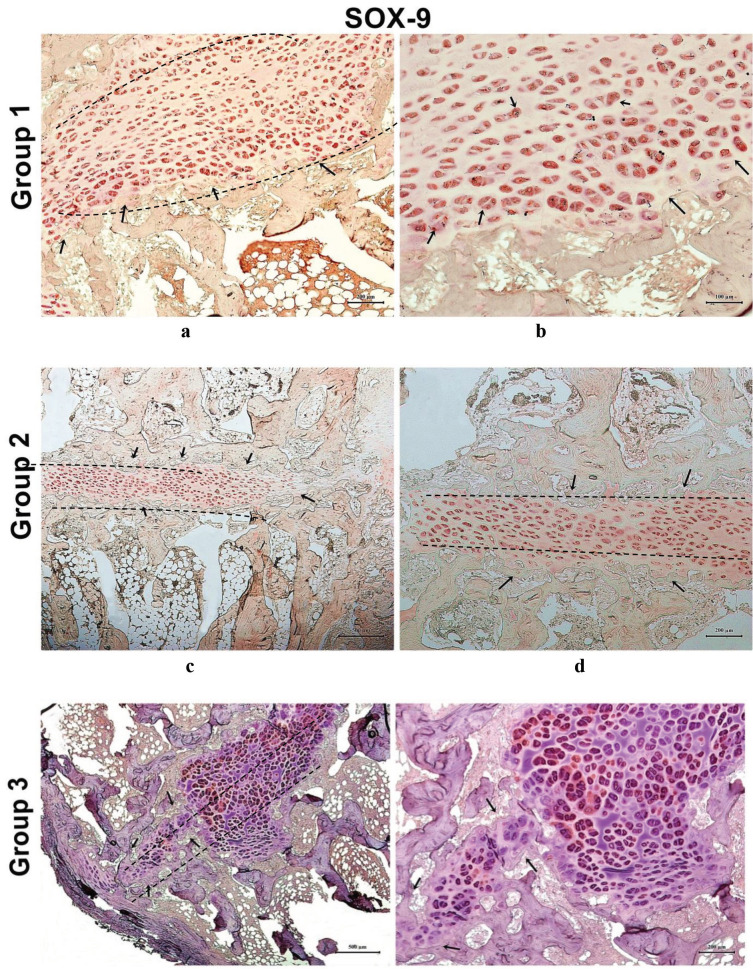
Sox-9 staining of tissue sections from all experimental groups. (a) Sections from Group 1 (a chondrocyte sheet was applied to proximal tibial physeal injury) revealed the prevention of bone bridge formation (black arrow) and the normal cartilaginous structure of the growth plate. Scale bar: 200 μm; (b) Higher magnification of the image in panel (a). Scale bar:100 μm; (c) Tissue sections from Group 2 revealed the differentiation of transplanted mesenchymal stem cell (MSC) sheets into chondrogenic cells by Sox-9 positivity and the prevention of bone bridge formation (black arrow). Scale bar: 500 μm; (d) Higher magnification of image in panel (c) Scale bar: 200 μm; (e) In Group 3, although Sox-9 positivity is observed, the cells are placed irregularly. Scale bar: 500 μm;(f ) Higher magnification of image in panel (e) Scale bar: 200 μm.Transversephyseal damage is indicated with a dashed line in the images in panelsa,c,d, and e.

**Figure 12:**
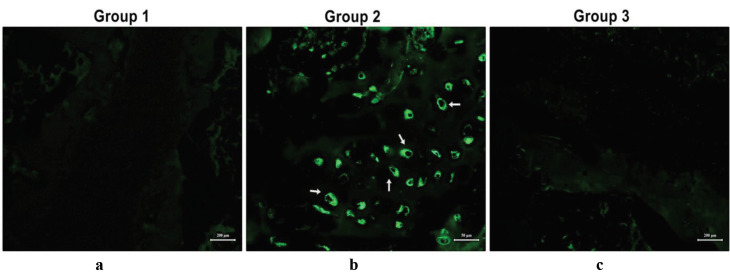
Green fluorescent protein (GFP) staining of sections from all experimental groups. (a) Group 1, chondrocyte cell sheet; (b) Group 2, mesenchymal stem cell (MSC) sheet; (c) Group 3, control group. GFP-positive MSCs are located in the damaged area (white arrow). Scale bars: a and c, 200 μm; b, 50 μm.

Sox-9 is a transcription factor that plays a key role in chondrocyte differentiation. While cells from Group 1 maintained their chondrogenic phenotype (Figures 11a and 11b), MSCs from Group 2 successfully differentiated into chondrocytes, as indicated by positive Sox-9 staining (Figures 11c and 11d). Moreover, when immunflourescent sections from all groups was analyzed comparatively for domains for cell migration and proliferation (Figures 12a–12c), the presence of GFP-positive RbBM-MSCs were observed in Group 2 in the damaged region by immunofluorescent staining in sections adjacent to H&E staining (Figure 12b).

**Figure 13:**
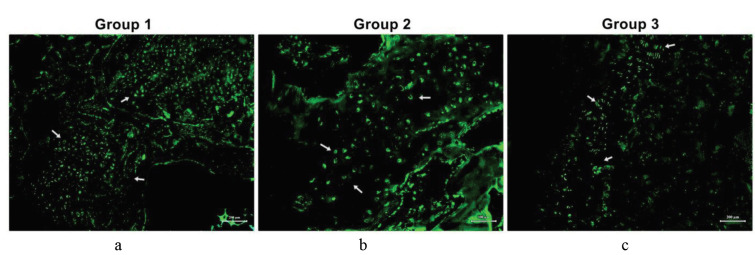
Aggrecan staining of sections from all experimental groups. (a) Group 1, chondrocyte cell sheet; (b) Group 2, mesenchymal stem cell (MSC) sheet; (c) Group 3, control. Scale bars: a and c, 200 μm; b, 100 μm.

Aggrecan is a cartilage-specific matrix proteoglycan used as a cartilage marker. When aggrecan staining analysis data of all experimental groups were investigated (Figures 13a–13c), no bridge formation was observed in rabbits with transplanted MSC sheets. Bone bridge formation was prevented by the transplanted MSCs, which exhibit aggrecan-positivity as proof of cartilaginous differentiation (Figure 13b).

## 4. Discussion

Treatment approaches for physeal cartilage arrest and related angular deformities in children as a result of trauma are not well established; stem cell transplantation is one of the most widely investigated methods for this condition. Although studies have demonstrated the success of allogeneic and autologous stem cell transplantation in the process of regenerating joint cartilage, very few studies have investigated the effect of stem cell transplantation on growth plate cartilage [23]. Gál et al. [24] and Hansen et al. [25] found that the formation of the bone bridge could not be prevented by using chondrocyte transfer to damaged tibial growth plates in sheep. However, Lee et al. [26] obtained chondrocytes from the iliac apophysis bone, prepared them in culture medium, and used them in an animal model in which iatrogenic growth plate damage had been induced. Although they reported successful results, their study was limited because of the use of a great part of the apophysis to achieve an adequate number of cells. Bentley et al. [27] stimulated new cartilage production in a damaged area of the growth plate via the transfer of allogeneic chondrogenic cells in New Zealand rabbits.

Although various studies have focused on MSC transfer in physeal damage owing to the reported limitations encountered in chondrogenic cell transplantation (e.g., the limited number of collectible cells and the duration of the culturing process), few studies have reported successful results. Gál et al. [24] performed allogeneic MSC transfers using hyaluronate/type 1 collagen/fibrin structural scaffolds following damage produced in the femoral distal epiphysis of rabbits. The authors reported that there were no signs of immunological rejection after 4 months and that hyaline cartilage of MSC origin developed in the damaged area. 

Furthermore, the results of allogeneic and autologous MSC treatments were compared in a femoral physeal injury model in New Zealand rabbits by Plánka et al. [28]. Measurements performed 4 months after the transplantation revealed that MSCs in both regions had differentiated into hyaline cartilage, and no significant differences were observed in the measurements of extremity lengths and valgus deformities in the distal femur. Thus, both autogenous and allogeneic MSC transplantations were noted to be effective in the treatment of physeal damage. However, as results of transplantation of 2 different types of allogeneic cells—MSCs and chondrocytes—under similar conditions have not been previously compared, the superiority of either method has not been clearly established.

The most important complication following physeal damage is the shortness of the injured extremity, owing to the cessation of growth. Although length differences existed between the operated and nonoperated tibias in all 3 groups in the present study, the distribution of differences in tibial length was significantly smaller in the chondrocyte transplantation group. The tibial lengths of the operated site were shorter than those of the contralateral site in all groups. However, the smaller difference between the lengths (median = 2.5 mm) in the chondrocyte groupand those in the other groups (median = 8.5 mm and 12.0 mm, respectively) shows that a functional physeal plate had formed and that the bone continued growing. Moreover, the MSC group exhibited significant improvement in the varus deformity over the control group, demonstrating the therapeutic efficacy of this treatment.

Histological staining clearly demonstrated bone bridge formation and fibrous tissue development in the area of physeal damage in the control group, while chondrocyte cells were found between the epiphysis and joint cartilage in the chondrocyte group, thereby preventing bone bridge formation. This finding suggests that the treatment was effective. The histological examination of the MSC group also demonstrated an arrest in bone bridge formation and the initiation of the differentiation of the transplanted cells to chondrocytes, further supporting the use of MSCs. Hence, both the measurements of deformity and the histological and immunohistochemical analyses suggest that the application of MSCs and chondrocytes are equally effective methods in the treatment of physeal arrest.

Providing the strongest possible microenvironment compatibility for transferred cells is still a challenge. We used cell sheet technology as a novel method for the first time with the aim of producing a functional growth plate without the need for previously published techniques, such as biodegradable scaffolds or growth factors [29–31].

An appropriate microenvironment is important to maximize chondrogenic efficacy in the region where the cells are transplanted. The expression levels of a chondrocyte transcription factor, Sox-9, was used as a reference to determine the suitability of the environment for chondrogenesis. Although Sox-9 staining was observed in tissue samples from all groups, the staining pattern was irregular in the control group samples, suggesting an ineffective endogenous repair response and, therefore, worse clinical outcomes. In the MSC and chondrocyte groups, Sox-9 staining patterns were distributed more regularly, suggesting that the chondrocytes in these samples could significantly arrest bone bridge formation.

Likewise, aggrecan is a cartilage matrix proteoglycan used as a cartilage marker. All 3 groups were positive for aggrecan staining, even though bone bridge formation was prevented only in the MSC and chondrocyte groups. While the formation of a functional growth plate, instead of a bone bridge in the damaged area, demonstrates the efficacy of the treatment applied, it is necessary to prove that the new growth plate developed from transplanted cells. Our observations of RbBM-MSCs, which were marked with GFP prior to transplantation, revealed that the transplanted cells continued to stay viable and proved that the cells began to create a functional growth plate.

However, the present study has some limitations, the first being the number of animals used in the study. Owing to the limited number of animals in each group, we could not euthanize the animals at different stages of the study and, therefore, did not monitor changes in histology at different stages following injury. Further studies with larger groups would be likely to more robustly demonstrate the healing capacity of the growth plate after treatment and yield more reliable results. Moreover, advanced radiological imaging techniques, such as microcomputed tomography, would be useful to image injured regions. We were not able to perform this technique, owing to limitations in the availability of radiological equipment. Additionally, achieving accurate alignment during radiographic imaging was a challenge. Coronal alignment measurements (i.e. MPTA) on radiographs could have been affected by the malposition of the limbs during imaging. To address this effect in the present study, the radiological imaging of both knees was performed simultaneously and with the assistance of the same surgeon to ensure the appropriate positioning of the animals each time. The animals were positioned in a straight dorsal recumbent fashion with gentle traction of the feet to secure the legs hyperextended and parallel.

Important factors in animal positioning were symmetry and the stabilization of the subject. The tibia was fixed in a straight position by gently extending the feet and then securing the legs and feet with tape onto the cassette. To optimize correct animal positioning, minor positional adjustments were sometimes applied to the abdomen. Although extensive attention was paid to these factors during imaging, some of the anteroposterior (AP) views demonstrated rotation. As we were not able to perform micro-CT imaging to determine the amount of rotation, the measurements were statistically analyzed. Although there seemed to be a rotation problem in AP views, the statistical analysis performed revealed that the distributions of both MPTA measurements and right tibial lengths were significantly different between the chondrocyte and control groups. While MPTA measurements could have been affected by the coronal alignment, the measurements of tibial lengths—which are not affected by rotational problems—were in agreement with MPTA measurements in all groups. In addition, we observed a shortening of the femur with increased valgus angulation in 2 of the subjects in the control group. Although the tibial side was the side where the operation was performed, excessive valgus angulation of the femur, with shortening of the extremity, can be considered to be a compensatory response to maintain joint congruity.

Another limitation of this study can be considered as small fraction CD45 and MHC Class II positivity during flow cytometry.

Rabbit bone marrow-derived MSCs (RbBM-MSCs) can be isolated using the methods previously described for human MSCs (hMSCs). RbBM-MSCs are considered to have similar characteristics to hMSCs and meet most standards set by the International Society for Cellular Therapy (ISCT). In a previous study, Dominici et al. proposed a minimum criteria to define RbBM-MSCs [32]. They summarized the minimum characteristics of RbBM-MSCs that should be used as a reference tool for future researchers in a table and also indicated that as new data or new technologies emerge, definitions or criteria should be made regularly.

However, these criteria should be sufficient as the basis for best defining RbBM-MSCs in terms of current knowledge status. It should be noted that the various differences between RbBM-MSCs and hMSCs should be considered; for instance, proliferation, osteogenic/chondrogenic expressions, and care should be taken when interpreting data from rabbits to humans. 

In a previous study, human and rabbit MSC surface markers were analyzed comparatively by flow cytometry. CD 34 and CD 45 markers were positive in RbBM-MSCs (17.4%) and negative in human MSCs [33].

Another study, by Kaiser et al., proved that MSCs obtained from bone marrow have a heterogeneous CD34 and CD45 phenotype under in vitro conditions [34]. This may explain the occurrence of CD45 positivity observed in RbBM-MSCs.

Based on these studies, we state that the phenotypic properties of MSCs originating from rabbit bone marrow may have changed during the culture process; also, due to the tissues being of rabbit origin, they are expected to express CD 45 and MHC class II positivity.

Nonetheless, our findings show that the cell sheet method could be used to effectively treat physeal arrest. In the present study, cell sheets were used for the first time to produce a functional epiphyseal growth plate without problems of durability or incompatibility, which has been shown to be common with structural scaffolds used in previous studies [27–29]. Indeed, the cell sheets developed in the present study facilitated growth plate regeneration and impeded the development of a bone bridge.

Further investigations, including immunohistochemical and long-term studies involving more advanced radiological tests, are needed to demonstrate the efficacy of such systems in humans. This would facilitate the design and development of cellular treatments for physeal arrest.

## Acknowledgments

The authors received funding from The Scientific and Technological Research Council of Turkey (TÜBİTAK-ARDEB) Research Support Programs Directorate. All financial support was provided by The Scientific and Technological Research Council of Turkey under the 3001 Starting R&D Projects Funding Programme (Project number: 114S152).

## Conflict of interest

The authors have no conflicts of interest to declare.

## Informed consent

Our request and the protocol to use laboratory animals received the approval from the Animal Ethics Committee of Kocaeli University with approval code: 10-5/2013.
